# Fatty Images of the Heart: Spectrum of Normal and Pathological Findings by Computed Tomography and Cardiac Magnetic Resonance Imaging

**DOI:** 10.1155/2018/5610347

**Published:** 2018-01-09

**Authors:** Giuseppe Cannavale, Marco Francone, Nicola Galea, Francesco Vullo, Antonio Molisso, Iacopo Carbone, Carlo Catalano

**Affiliations:** ^1^Department of Radiological, Oncological and Anatomo-Pathological Sciences, Policlinico Umberto I, Sapienza University of Rome, Viale Regina Elena 324, 00161 Rome, Italy; ^2^Servizio Radiologia, Casa di Cura San Michele, Via Appia 190, 81024 Maddaloni, Italy

## Abstract

Ectopic cardiac fatty images are not rarely detected incidentally by computed tomography and cardiac magnetic resonance, or by exams focused on the heart as in general thoracic imaging evaluations. A correct interpretation of these findings is essential in order to recognize their normal or pathological meaning, focusing on the eventually associated clinical implications. The development of techniques such as computed tomography and cardiac magnetic resonance allowed a detailed detection and evaluation of adipose tissue within the heart. This pictorial review illustrates the most common characteristics of cardiac fatty images by computed tomography and cardiac magnetic resonance, in a spectrum of normal and pathological conditions ranging from physiological adipose images to diseases presenting with cardiac fatty foci. Physiologic intramyocardial adipose tissue may normally be present in healthy adults, being not related to cardiac affections and without any clinical consequence. However cardiac fatty images may also be the expression of various diseases, comprehending arrhythmogenic right ventricular dysplasia, postmyocardial infarction lipomatous metaplasia, dilated cardiomyopathy, and lipomatous hypertrophy of the interatrial septum. Fatty neoplasms of the heart as lipoma and liposarcoma are also described.

## 1. Introduction

**Figure 1:**
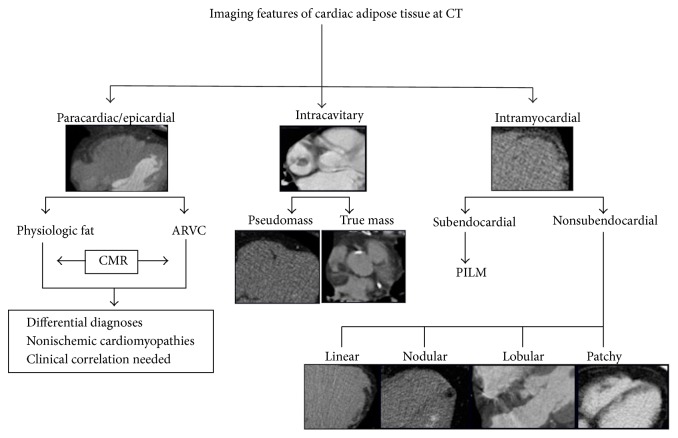
Imaging features of cardiac adipose tissue by computed tomography. CT: computed tomography; ARVC: arrhythmogenic right ventricular dysplasia; CMR: cardiac magnetic resonance; PILM: postmyocardial infarction lipomatous metaplasia.

Ectopic intracardiac fatty images are not uncommon findings in thoracic imaging in both healthy and diseased patients. Development of diagnostic techniques with high spatial and contrast resolution as computed tomography (CT) and cardiac magnetic resonance (CMR) enabled detailed fat visualization within the heart. An incidental frequency of cardiac adipose images in about 11% of the patients performing cardiovascular CT examinations has been estimated [1]. However foci of ectopic cardiac adipose tissue could sometimes be not pointed out in radiological reports, even in pathological conditions, mostly if the exam is not specifically focused on the heart. This pictorial review has the aim of illustrating CT and CMR features of cardiac fatty images, with the purpose of assisting in recognizing their typical imaging appearances. A selection of the most common characteristics of cardiac fat in a spectrum of normal and pathological conditions will be depicted, providing a simplified step-by-step approach to discriminate between various imaging patterns, outlined by the scheme in Figure 1. Different imaging characteristics between physiologic and pathologic intracardiac fat are summarized in Table 1, readapted from Kimura et al. [2].

## 2. Nonpathological Ectopic Cardiac Fat

**Figure 2:**
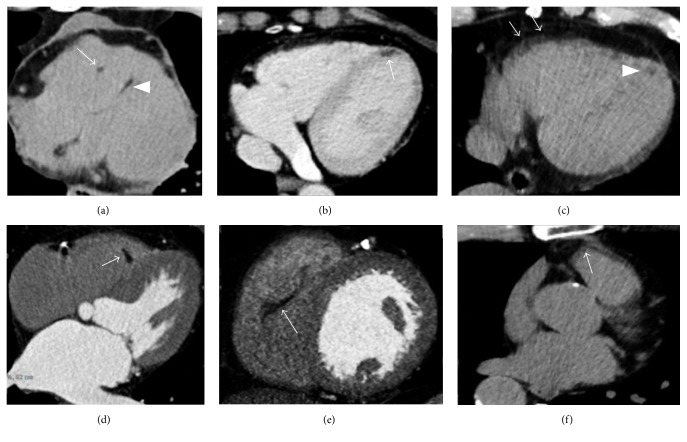
Typical incidental physiologic cardiac fatty images detected by CT. (a) Small fatty foci are seen in right ventricular moderator band (arrow) as within the interventricular septum (arrowhead) and in the left ventricular myocardial apex ((b) arrow). A small amount of pericardial fluid is also noted in (a). (c) A linear shaped adipose infiltration is, respectively, illustrated in the right ventricular free wall (arrows) as in the left ventricular apex (arrowhead), within the right ventricular moderator band ((d) arrow), in the right ventricular trabeculae ((e) arrow), and at the right ventricular outflow tract ((f) arrow).

**Figure 3:**
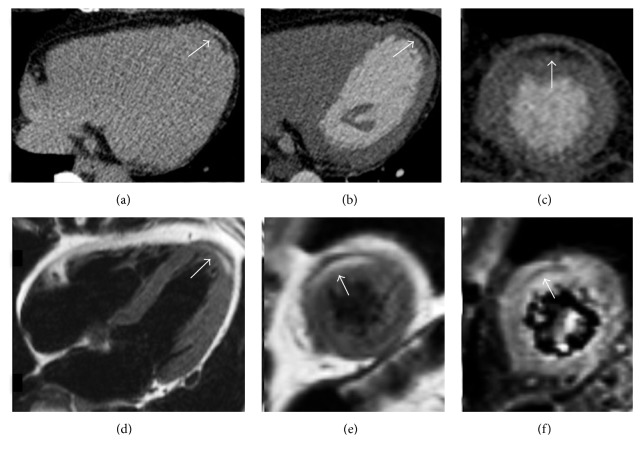
Thoracic noncontrast CT axial image in a 45-year-old female without any cardiac disease incidentally reveals a curvilinear hypodense stripe located within the subepicardial layer of the myocardium at the left ventricular apex, with negative attenuation values (a). The finding is successively confirmed at a cardiac CT postcontrast medium administration in axial view ((b) arrow) and in short axis multiplanar reconstruction ((c) arrow). In (d–f), the corresponding CMR exam, requested for a further evaluation, confirms the presence of the hyperintense stripe at the same place on T1-weighted black blood in four chambers ((d) arrow) and in short axis view ((e) arrow), with associated nulled signal on fat-suppressed T2-weighted image ((f) arrow), according to physiologic left ventricular apical fatty tissue.

Physiologic ectopic cardiac adipose tissue may normally be present in healthy adults not affected by any cardiac disease, without clinical consequences [3]. In fact ectopic intramyocardial fat extending from the epicardiac adipose tissue is a relatively common incidental finding during a routine chest or cardiac CT, usually more frequent in the right ventricle (RV) than in the left ventricle (LV), with an overall prevalence of RV ectopic intramyocardial fat of 16–43%, [3]. In a series of autopsies, RV cardiac adipose tissue was detected in 85% of patients free of cardiac diseases [4]. Especially in the elderly, CT may commonly demonstrate physiologic RV myocardial fat with linear or patchy morphology located in the free wall, in the subepicardial layers of anterolateral or apical segments, and in the RV outflow tract (RVOT), together with a preserved myocardial thickness [5]. However in some cases when RV fatty infiltration is more prominent it can also extend from the epicardial region through the myocardium until the subendocardial layer, with consequent increase of the myocardial wall thickness [6]. Small amounts of physiological fat can also be routinely detected in other typical locations such as the RV and LV trabeculae, RV moderator band, and interventricular septum as well as in the LV apex (Figures 2 and 3). Frequency and degree of RV ectopic intramyocardial fat increase with age, being considered as a part of the aging process, while its relationship with obesity remains still unclear [7]. When intracavitary and circumscribed in morphology, nonpathological adipose fatty foci may sometimes assume a pseudomass appearance, resembling tiny cardiac tumors, as, for example, in the adipose degeneration of the RV moderator band: in this eventuality, knowledge of the most common locations of physiologic cardiac fat and correlation with clinical data are essential key points helping to orientate toward a correct interpretation.

## 3. Arrhythmogenic Right Ventricular Cardiomyopathy

**Figure 4:**
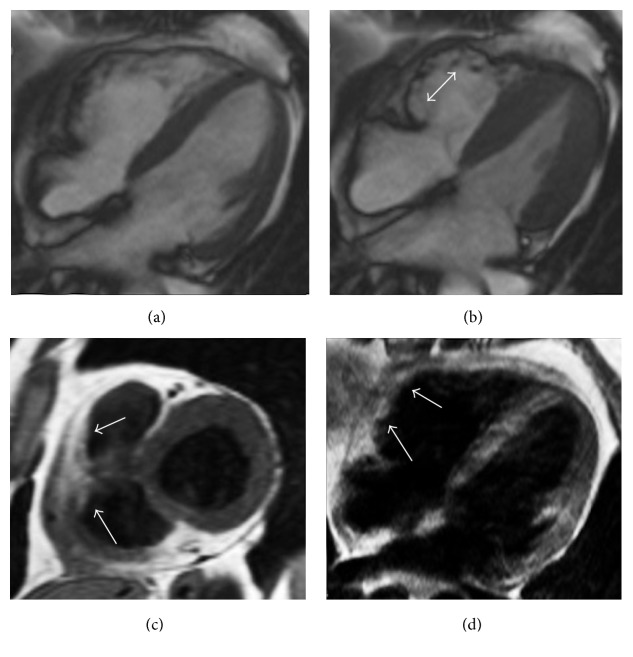
Arrhythmogenic right ventricular cardiomyopathy in a 27-year-old man presenting with palpitations. Cine-SSFP four-chamber images, respectively, in diastole (a) and systole (b) demonstrate an aneurysmatic aspect with dyskinesia of the right ventricular free wall that appears corrugated ((b) double-headed arrow). T1-weighted black blood sequences in short axis (c) and four-chamber (d) views demonstrate adipose infiltration phenomena in the right ventricular free wall (arrows).

**Figure 5:**
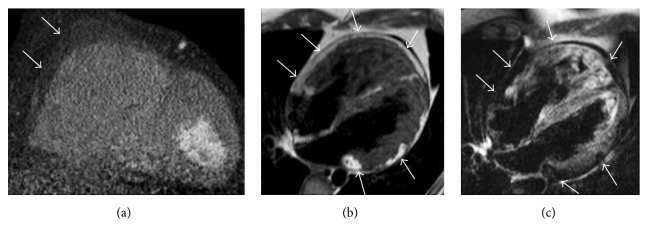
Biventricular arrhythmogenic ventricular cardiomyopathy in a 32-year-old man presenting with syncope and familiar history of sudden cardiac death. Cardiac CT with multiplanar reconstruction in short axis plane (a) reveals right ventricular enlargement with diffuse fatty infiltration of the right ventricular free wall (arrows). CMR exam in the same patient with T1-weighted black blood in four-chamber plane (b) illustrates multifocal adipose infiltration of the right ventricular free wall, of the right ventricular apex, and within the left ventricular lateral wall (arrows). A four-chamber black blood T2-weighted image with fat suppression confirms the biventricular myocardial adipose infiltration, with corresponding low intensity signal in the same locations (arrows).

Arrhythmogenic right ventricular cardiomyopathy (ARVC) is an inherited cardiac disease characterized by structural and functional abnormalities that may lead to arrhythmias and sudden cardiac death [8]. Pathologic changes of this disease are mostly represented in the RV, comprehending ventricular dilatation, thinning of the free wall, microaneurysms, and myocardial fibrofatty replacement phenomena [9]. Particularly ectopic intramyocardial adipose tissue in ARVC consists of a fibrolipomatous infiltrate that is usually placed along the RV free wall in a prevalent subepicardial distribution [10]. Adipose myocardial infiltration may generally also take place in the so-called triangle of dysplasia, a region comprehended between the RV inflow tract, the RVOT, and the RV apex [11]. Fibrofatty replacement in ARVC consists of a wave-front phenomenon starting from the epicardium and extending to the endocardial layer, with the latter that is generally spared (Figure 4). However there are histologic evidences of fibrolipomatous metaplasia not only in the RV, but also in the LV lateral wall in 47%–76% of cases and in the interventricular septum in 20%, with a predilection for the posterolateral and posteroseptal segments [7]. Notably LV fatty involvement often occurs in the later stage of the disease following the RV adipose infiltration, although some cases of isolated LV disease have been previously reported (Figure 5) [12]. However myocardial fibrofatty infiltration at imaging is currently not included in the revised criteria for the diagnosis of ARVC [13]. In fact, despite CMR being actually considered the more suitable noninvasive method for recognizing myocardial fatty replacement, differentiating myocardial from epicardial fat may be challenging due to CMR limited spatial and contrast resolution. In order to overcome this issue, previous studies performed with CMR suggested the use of Turbo Spin Echo T1-weighted black blood sequences with and without fat saturation pulses to facilitate the differentiation between pathological fatty infiltration from normal epicardial adipose tissue [14]. Late gadolinium enhancement (LGE) technique also demonstrated to be helpful in detecting myocardial fibrous degeneration, although it may be difficult to distinguish enhancement from intramyocardial fat located in a thinned RV myocardium, because both appear with hyperintense signal [15]. Furthermore CMR particularly represents the better technique to differentiate a fibrolipomatous infiltration in ARVC from physiological fat, despite this being not always straightforward because they both present with linear or patchy morphology and spare the subendocardium: however in ARVC fatty infiltration is usually present in a RV free wall that is considerably thinned (generally <2 mm), while in nonpathological adipose myocardial infiltration the wall thickness is normally preserved or even increased. Finally other nonischemic cardiomyopathies should also be included in the differential diagnosis with ARVC in presence of adipose tissue not involving the subendocardial layer of the myocardium: the correlation with clinical data, the elderly age, and the absence of the other CMR diagnostic criteria for ARVC should more likely orientate to exclude this disease.

## 4. Postmyocardial Infarction Lipomatous Metaplasia

**Figure 6:**
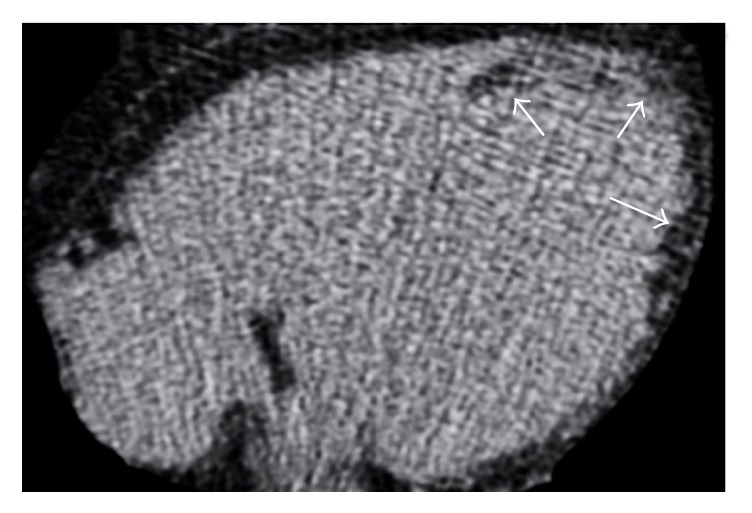
Cardiac CT axial image without contrast media administration in a 72-year-old male after an extensive chronic myocardial infarction shows the presence of a curvilinear hypodense stripe (arrows) with negative attenuation values (−20 Hounsfield units), located within the subendocardial layer of the left ventricular apex extending also to the left ventricular lateral wall, findings related to a postischemic lipomatous metaplasia.

**Figure 7:**
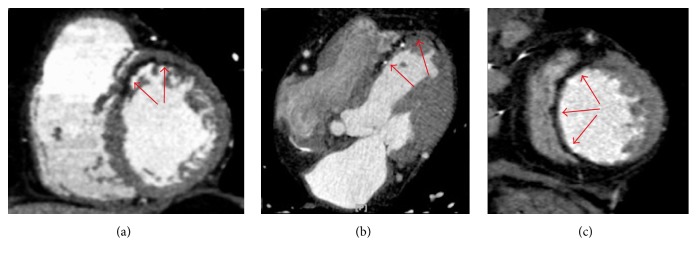
Cardiac CT after contrast medium administration in three different patients with postinfarction lipomatous metaplasia. (a) demonstrates a curvilinear hypodense fatty stripe with subendocardial distribution located in the anterior segment of the left ventricular myocardium in a short axis multiplanar reconstruction (red arrows). A similar finding is present in axial view (b) in the interventricular septum and left ventricular apex, also associated with tiny calcifications and wall thinning (red arrows). In (c) a lipomatous metaplasia involving the whole interventricular septum together with remarkable corresponding wall thinning is illustrated on a short axis multiplanar reconstruction (red arrows).

Postmyocardial infarction lipomatous metaplasia (PILM) is a tissue transformation process that may take place within the scar region after a healed myocardial infarction (MI). The prevalence of PILM at histology in the LV reached values of 68–84% of excised heart transplanted for ischemic heart disease [16]. In imaging studies the prevalence of PILM among patients with history of LV MI ranged, respectively, from 22%–62% with CT to 68% with CMR [17, 18]. PILM correlates with elderly age, male gender, and conditions related to reperfusion therapy as percutaneous coronary artery intervention and placement of coronary artery by-pass grafts [18]. This process is related to different factors as the inability of necrotized myocytes to metabolize free fatty acids, transdifferentiation of myocytes in adipocytes after reperfusion therapy, and impaired regional myocardial wall contractility [18]. Fatty myocardial metaplasia tends to increase with time since MI, being usually seen in patients presenting with a chronic MI, more than 6 months from the acute ischemic event [19]. In fact it is known that adipocytes are histologically preceded by fibrous scar tissue onset; although it was noted that their presence begins early after MI, they are not immediately detectable due to the limited sensitivity of the current available imaging techniques [19]. PILM generally does not present a transmural extension, generally involving <75% of the myocardial thickness and always originating from the subendocardial layer. A study performed with CMR demonstrated that LV intramyocardial fat volume in PILM is significantly related to parameters as MI volume, LV ejection fraction, LV end-systolic, and end-diastolic volume indexes [20]. The typical aspect of PILM in CT consists of curvilinear hypodense stripes with negative attenuation values (−20 Hounsfield units) located within the subendocardial layer, often associated with myocardial wall thinning and sometimes also with calcifications, confinated in a coronary artery perfusion territory (Figures 6 and 7). At CMR imaging PILM is generally detectable as subendocardial hyperintense strikes both in T1-weighted black blood and in T2-weighted bright blood sequences, slightly less hyperintense in cine-SSFP imaging, with nulled signal in fat suppression sequences. Furthermore PILM is usually disposed within a thinned necrotic myocardial wall that presents contraction abnormalities as dyskinesia or hypokinesia. However despite an accurate evaluation at CMR, it may be challenging to clearly differentiate PILM located in the RV from a physiologic fat infiltration only on the base of the current imaging findings, due to the normally thinner aspect of the RV free wall. Recently CMR with T1-mapping technique by Sh-MOLLI sequences have been proposed to detect PILM in patients with chronic MI, reporting increased native-T1 values in the infarcted region compared to remote myocardium, maybe related to the presence of fat [21]. Although it is not clear how these findings could have been affected by PILM, this suggests that T1-mapping technique may have the potential to detect small foci of intramyocardial fat even in an early stage after MI.

## 5. Dilated Cardiomyopathy

**Figure 8:**
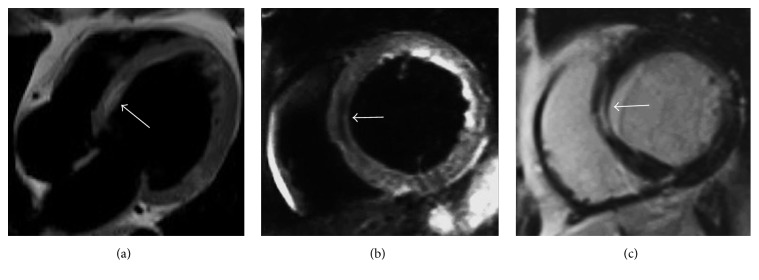
CMR examination in a 62-year-old woman with idiopathic dilatative cardiomyopathy. Four-chamber T1-weighted black blood image (a) demonstrates left ventricular chamber enlargement with an intramyocardial hyperintense stripe in the interventricular septum. Short axis black blood T2-weighted with fat suppression (b) shows a signal drop of the stripe, confirming the presence of intramyocardial fat. On short axis late gadolinium enhancement T1-weighted sequence (c) the adipose tissue location corresponds to myocardial enhancement due to concomitant intramyocardial fibrosis with mesocardial distribution within the interventricular septum.

Idiopathic or “primary” dilated cardiomyopathy (DCM) is characterized by left or biventricular dilatation and impaired systolic function without significant coronary artery lesions, excluding other possible etiologic causes [22]. Primary forms represent about 30–40% of cases, while secondary subtypes of DCM derive from a heterogeneous group of affections including ischemic, inflammatory, autoimmune, toxic, or metabolic disorders [22]. Specific hallmarks of DCM at histology include hypertrophy and degeneration of the myocytes, interstitial fibrosis, intramyocardial cluster of lymphocytes, and finally fibrofatty infiltration [23]. Intramyocardial fat deposition is a relatively common phenomenon in DCM, resulting in about 18–24% of cases [24]. The pathogenesis of fatty metaplasia in DCM is still unknown, but intramyocardial fibrosis could probably be the precursor of adipose tissue, such as mentioned above in PILM. However in primary DCM this process may be rather histologically related to the apoptotic phenomena of myocardial myocytes and interstitial cells, with a prevalence of lipomatous metaplasia reported until 26% of myocardial fibrosis areas from an autopsy study [25]. CMR has the capability to detect both myocardial fibrosis and intramyocardial fatty deposition: notably in DCM the typical location of adipose tissue usually follows myocardial fibrosis, being represented by curvilinear fatty stripes with mesocardial distribution detectable within the LV myocardium (Figure 8). Furthermore a study involving 85 DCM patients reported a prevalence of concomitant fat deposition and enhancement of 8% of the patients, enhancement without accompanying fat of 18%, and sole fat deposit without enhancement of 1%: these data strengthen the association between combined fibrosis and adipose tissue metaplasia [26]. However distinguishing fibrous tissue from fat in LGE sequences may be difficult even at CMR, because both present with high signal. Lu et al. proposed a fat-water separation imaging in DCM to distinguish fat from fibrosis at LGE imaging, moreover pointing out that intramyocardial fat volume was significantly related to LV global function [27]. These findings may suggest that adipose deposition in DCM could be an indicator of a worse prognosis.

Regarding the differential diagnoses of this disease, left-dominant ARVC with fibrofatty replacement of the LV represents an affection hardly distinguishable from idiopathic DCM at CMR imaging: in this case a careful evaluation of the adipose tissue and of the LGE location is crucial, being both in ARVC predominantly represented in the subepicardium and not in the middle layer of the myocardium as in primary DCM.

Besides the evidence of fatty intramyocardial tissue at imaging sparing the subendocardial layer and having no relationship with a coronary artery perfusion territory allows excluding a DCM secondary to a PILM.

**Figure 9:**
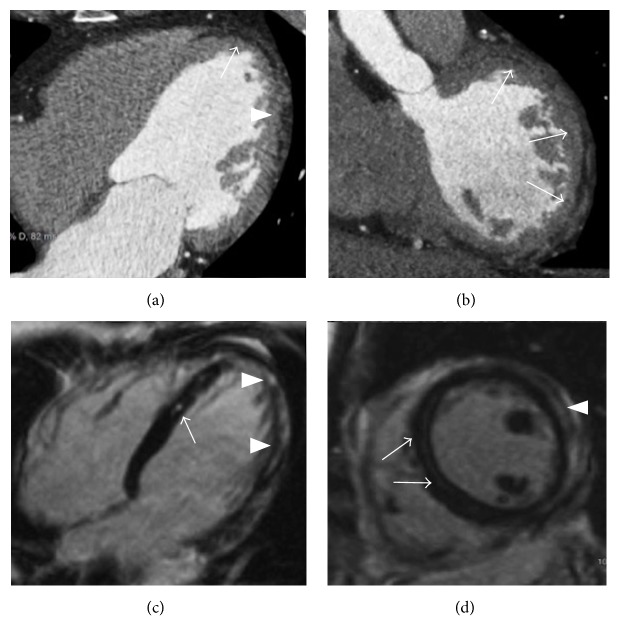
Postinflammatory lipomatous metaplasia in a 52-year-old man at 6 months from an acute viral lymphocytic myocarditis. Cardiac CT in axial view (a) illustrates linear hypoattenuating stripes with negative attenuation values placed in the subepicardial layer of the left ventricular myocardium, involving the apex (arrow) and the lateral wall (arrowhead). The lateral wall fatty involvement is evident also in short axis multiplanar reconstruction ((b) arrows). A mild enlargement of left ventricle is also noted (a). CMR in the same patient with late gadolinium enhancement T1-weighted sequences, respectively, in four-chamber (a) and short axis (d) planes shows a patchy and curvilinear enhancement in the interventricular septum (arrows), left ventricular apex, and lateral wall (arrowheads), sparing the subendocardial layer and not following a coronary artery perfusion territory, findings compatible with a postinflammatory damage.

Finally fatty myocardial metaplasia phenomena in secondary DCM have also been described after an inflammatory process as chronic myocarditis (Figure 9) [28]. In these patients ventricular dilatation may occur in the late stage after inflammation as a consequence of a direct damage of the myocytes by the etiologic agent causing extensive myocardial injury [29]. This process may lead to a DCM pattern of presentation with associated adipose metaplasia of the damaged myocytes, probably hypothesizing the same histological mechanisms previously described in PILM.

## 6. Lipomatous Hypertrophy of the Interatrial Septum

**Figure 10:**
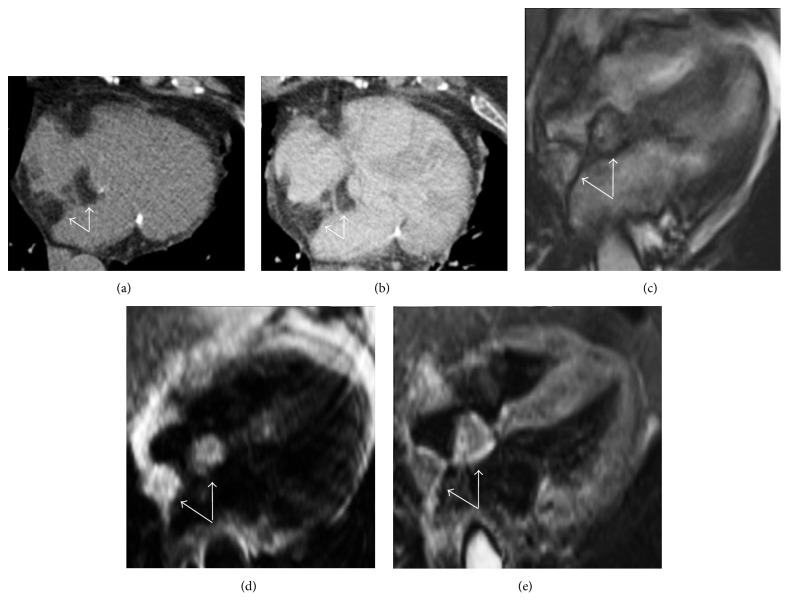
Lipomatous hypertrophy of the interatrial septum as incidental finding in an asymptomatic 72-year-old female. CT imaging of the heart in precontrast scan (a) and after contrast medium administration (b) in axial views demonstrates a bilobular dumbbell shape hypoattenuating lesion with lobular morphology located within the interatrial septum that appears thickened (arrows). CMR examination requested for a further evaluation confirms the presence of a bilobular mass with corresponding hyperintensity in four-chamber cine-SSFP sequence surrounded by the typical black boundary artefact ((c) arrows) as in T1-weighted black blood image ((d) arrows), with low signal on black blood T2-weighted sequence with fat suppression (arrows).

**Figure 11:**
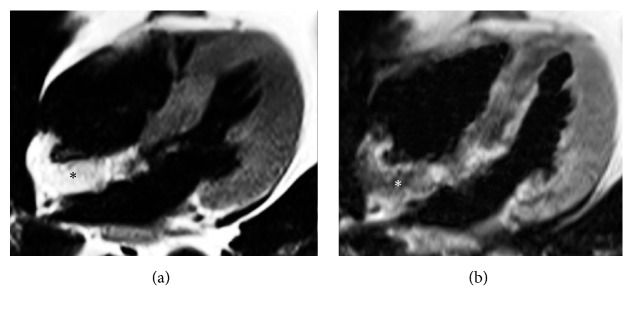
CMR exam in a lipomatous hypertrophy of the interatrial septum in an asymptomatic 68-year-old female. Four-chamber black blood T1-weighted image (a) shows a hyperintense lobular mass infiltrating the interatrial septum and part of the posterior wall of the right atrium (asterisk). Corresponding T1-weighted image with fat suppression (b) demonstrates homogeneous signal drop of the mass, confirming the presence of adipose tissue (asterisk).

Lipomatous hypertrophy of the interatrial septum (LHIAS) is an uncommon benign disorder with fatty accumulation into the interatrial septum that usually measures a transverse diameter > 2 cm in this condition [30]. LHIAS is histologically characterized by adipocytes hyperplasia with fat infiltration between the myocardial fibers of the interatrial septum [31]. The overall prevalence of this condition in the general population is estimated to be about 1–8%, being more frequent in the elderly and obesity, with a higher incidence reported in women [32]. LHIAS is asymptomatic in the majority of cases, but it may also be related to supraventricular cardiac arrhythmias, P-wave alterations on electrocardiogram, pericardial effusions, and sudden cardiac death [32]. Being mostly asymptomatic, this condition is generally diagnosed incidentally at imaging, surgery, or autopsy [32]. Its usual imaging appearance in CT or CMR is characteristic, consisting of a typical smooth dumbbell-shaped fatty mass with lobular pattern of presentation, located within the interatrial septum (Figures 10 and 11). Notably LHIAS typically spares the fossa ovalis and does not show contrast enhancement.

## 7. Other Diseases

**Figure 12:**
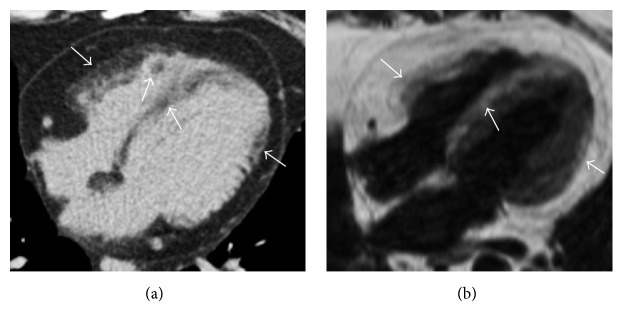
Axial thoracic noncontrast CT scan (a) shows multiple biventricular cardiac fatty foci with patchy pattern of appearance (arrows) in a patient with TSC, findings noted during an exam requested to evaluate pulmonary lymphangioleiomyomatosis. CMR with T1-weighted black blood four-chamber image (b) in the same patient confirms the presence of multiple corresponding fatty areas within the myocardium showing hyperintense signal (arrows).

*Tuberous sclerosis complex* (TSC) represents a genetic multisystemic disorder with cardiac tumor-like manifestations such as rhabdomyomas that regress during childhood and intramyocardial fat-containing lesions in adults [33]. Fatty images within the heart in TSC are often incidentally described during chest CT requested to evaluate pulmonary lymphangioleiomyomatosis, a lung disease associated with TSC [34]. A previous study found an incidence of fatty intramyocardial foci of 64% in a group of 55 TSC patients evaluated by CT [35]. Although multiple intramyocardial lipomas (LP) have been associated with TSC, it remains uncertain if fatty foci actually represent true LP [35]. A possible relationship between the presence of renal angiomyolipomas and cardiac fat-containing lesions in TSC has also been hypothesized, considering fat lesions as cardiac localizations of angiomyolipomas [36]. Notwithstanding the natural history of these fatty foci and their potential clinical implications are still not clear, their presence currently being not included in the established diagnostic criteria for TSC [37]. However in patients presenting with multiple focal intramyocardial fat depositions detected at imaging, possible manifestations of TSC in other organs should be investigated. In this disease both CT and CMR usually demonstrate circumscribed fatty foci or present with a patchy appearance, mainly located in the interventricular septum and in the LV lateral wall (Figure 12). Notably adipose lesions in TSC generally do not show an invasive behaviour or contrast enhancement, even when large in dimensions [38].

*Muscular dystrophies* (MD) are X-linked recessive diseases characterized by cardiac involvement with replacement of the myocardium by connective and adipose tissue [39]. Particularly in Duchenne and Becker muscular dystrophies the myocardial regions most extensively affected are the LV inferobasal and later wall, with sparing of the RV and the atria [40]. Typical manifestations of MD consist of LV dysfunction and heart failure, although the presence of ectopic intramyocardial fat in MD has never been reported with current imaging techniques, being revealed only by histology [40].

*Hypertrophic cardiomyopathy* (HCM) has also been described to be related to the presence of intramyocardial fatty foci, with adipose tissue placed within the thickened myocardial segments involved by this disease [41]. The real incidence and underlying causes of fatty myocardial metaplasia in HCM are actually unknown; however ectopic intramyocardial fatty foci in patients with a thickened LV wall reported an incidence of 11% performing thoracic CT [9].

Although the presence of ectopic adipose tissue within the myocardium is often detectable in CT and CMR images, the “cardiac steatosis” defined as cardiomyocytes accumulation of lipids is not easily appreciable with these imaging techniques. Cardiomyopathies secondary to lipid metabolism disorders may have a heterogeneous clinical expression, mimic dilated or hypertrophic cardiomyopathy, and led to progressive heart failure.

*Fabry disease* is a rare X-linked genetic lysosomal storage disease, resulting from dysfunctional metabolism of sphingolipids [42].

Intramyocardial triglyceride deposition is linked to various pathological hereditary and acquired conditions (e.g., diabetes mellitus, metabolic syndrome, and obesity).

*Triglyceride deposit cardiomyovasculopathy* is a genetic disorder with accumulation of triglyceride in cardiomyocytes and smooth muscle cells due to abnormal intracellular deposit of triglyceride and its substrates [43, 44].

Usually in these conditions myocardial signal is not altered in the conventional T1- and T2-weighed sequences, whereas T1 mapping technique has emerged as a sensitive and specific CMR biomarker of tissue lipid accumulation irrespective of ventricular morphology and function [45].

Moreover a case has been reported where the excessive production of fatty acids in subjects with high alcohol consumption caused an alcohol-related cardiomyopathy associated with left ventricular fatty infiltration [46].

## 8. Lipoma

**Figure 13:**
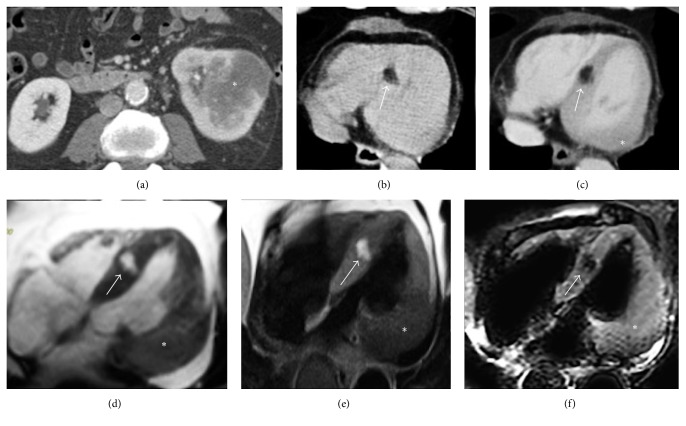
Interventricular septal lipoma incidentally detected in a 79-year-old male with concomitant left renal cancer on a staging total body CT exam ((a) asterisk). During thoracic CT scan a hypoattenuating nodular-shaped lesion with negative attenuation values located in the interventricular septum is incidentally noted before ((b) arrow) and after contrast medium administration ((c) arrow). A renal cancer metastatic lesion in the lateral wall of the left ventricle ((c) asterisk) is also present, together with moderate pericardial fluid. CMR in four-chamber views in the bottom line images confirms the nodular area placed within the interventricular septum on cine-SSFP sequences that appears hyperintense and delimited by the characteristic black boundary artefact ((d) arrow), with hyperintense signal also on T1-weighted black blood sequence ((e) arrow) and hypointense appearance on T2-weighted black blood with fat suppression ((f) arrow). These findings are related to an interventricular septal lipoma ((b)–(f) arrow) with a concomitant left ventricular renal cancer metastatic lesion ((c)–(f) asterisk), both histologically proven.

LP represent the second most common benign cardiac tumor after myxomas, accounting for about 10% of primary cardiac neoplasms [47]. They are often discovered incidentally but in rare cases they can become symptomatic due to obstruction of blood flow or compression of the ventricular chambers. The majority of LP are in extramyocardial location although they can also be intramyocardial, arising in any cardiac chamber and within the pericardial space, at any age [48]. These tumors are generally slow growing but, depending on their location, may, respectively, rarely cause compression of the coronary arteries or of the pericardial space when placed in the subepicardial layer, outflow obstruction when located in the subendocardium, or arrhythmias when intramyocardial [49]. Most are solitary tumors occupying the right atrium or the LV chamber, but multiple cardiac LP can also occur and have been described in patients with TSC, although it is not currently established if fat lesions in TSC histologically represent true LP [50]. In CT imaging LP are fat attenuating, well-circumscribed, and encapsulated masses showing a nodular pattern of appearance, without contrast enhancement. At CMR examination LP generally show a homogeneous nodular high signal intensity on T1-weighted images, slightly less hyperintensity on T2-weighted sequences, and suppression on fat-saturated images, similar to subcutaneous or mediastinal fat (Figure 13). When LP are intramyocardial they are usually seen on cine-SSFP image as high-signal-intensity nodular areas surrounded by a black boundary artefact, normally present at muscle-fat interface [51]. Sometimes internal thin septations could be noted, while they are not enhanced after contrast material administration. Differentiation between LP and LHIAS is generally not so difficult, with the latter manifesting as an adipose unencapsulated bilobular mass located in the interatrial septum showing a characteristic lobular dumbbell shape, sparing the fossa ovalis.

## 9. Liposarcoma

Primary cardiac liposarcomas (LS) are extremely rare neoplasms, mostly originating from the right chambers of the heart, particularly from the right atrium [52]. Cardiac LS usually remain undiagnosed until symptoms of infiltration of the surrounding anatomical structures develop, such as chest pain and dyspnea or signs of congestive heart failure in the late stage [53]. The most common histological subtype of LS is the well-differentiated subtype (or “lipoma-like” due to a higher amount of fat content: 40–50% of cases), but they can also present as myxoid, round-cell, or pleomorphic [54]. The well-differentiated and the myxoid subtypes generally show local aggressive behaviour, while the latter two subtypes early metastasize to the lungs and frequently recur locally after surgical resection [55].

Cardiac metastases from a primary LS located in other anatomical districts have also been reported, although rare [56]. In fact LS represents the second most common tumor producing cardiac metastases after malignant histiocytoma among soft-tissue neoplasms, with about 10–18% of all cases [57]. Particularly myxoid variant LS constitutes the most frequent histological subtype with metastatic potential to the heart (about 30–50% of cases) [57].

**Figure 14:**
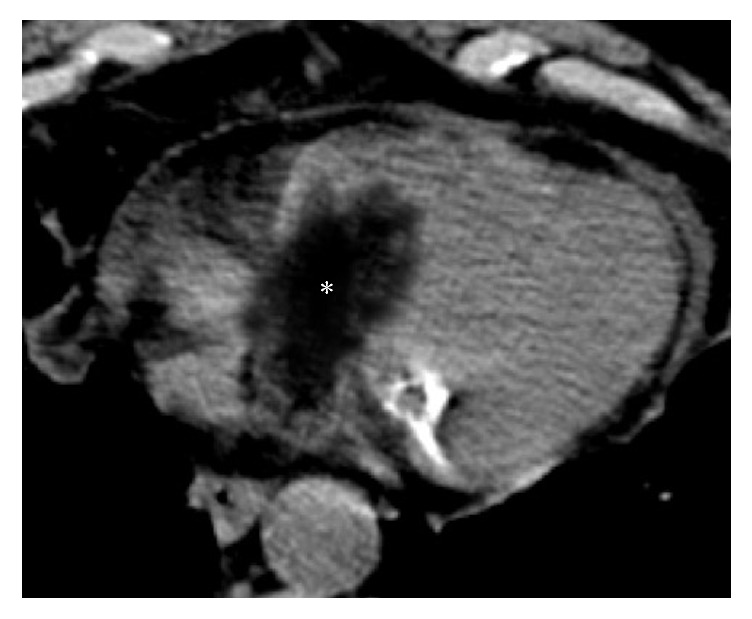
Histologically proven cardiac primary well-differentiated liposarcoma incidentally detected on a CT scan in a 73-year-old patient presenting with chest pain and dyspnea. The noncontrast CT axial image shows a hypoattenuating solid mass (asterisk) with prevalent negative densitometric values, inhomogeneous content, and irregular margins, extending from the right atrium into the right ventricle through the tricuspid valve, as for an infiltrative behaviour. A mild amount of pericardial fluid is also noted.

Typical features in CT imaging of cardiac primary LS or its metastatic lesions consist of large unencapsulated hypoattenuating inhomogeneous masses, mostly rounded shaped with thick septa and mild contrast enhancement, infiltrating the surrounding cardiac structures and the pericardium (Figure 14). Notably LS may have different patterns of attenuation in CT depending on the amount of fat and combined soft-tissue components within the tumor. CMR is able to better define the infiltration of the surrounding structures and allows evaluating possible functional damage on vascular and valvular components of the heart. In terms of differential diagnosis, it could sometimes be difficult to distinguish well-differentiated LS from LP based only on the current imaging techniques, since moreover both have a nodular pattern of appearance. However LS, unlike LP, are usually unencapsulated and have infiltrative behaviour and contrast enhancement. Other findings more orientating toward LS comprehend an inhomogeneous appearance despite the presence of an intralesional fatty portion, larger dimensions, and a more prominent soft-tissue component.

## Figures and Tables

**Table 1:** Differentiating features between physiologic and pathologic cardiac fat.

Type of fat	Patient's characteristics	Cardiac location	Intramyocardial distribution	Morphological imaging pattern	Myocardial thickness	Ventricular size
Physiologic	Elderly, also without any cardiac disease associated	More in the RV than in LV: RV free wall, RVOT, RV and LV trabeculae, RV moderator band, interventricular septum, RV apex	Mostly subepicardial, sometimes with full thickness	Linear or patchy	Normal or thickened	Normal
ARVC	Young to middle age, male dominant	RV free wall, RVOT, RV apex, interventricular septum, LV lateral wall	Mostly subepicardial	Linear or patchy	Thinned	Enlarged RV, enlarged LV in left-dominant disease
PILM	Elderly or middle age, generally male dominant, within a chronic MI	Usually in LV myocardium, within a chronic MI scar, in a coronary artery irroration territory	Mostly subendocardial	Linear	Thinned, sometimes with calcifications	Normal or enlarged LV
DCM	Elderly or middle age	In LV myocardium, following myocardial fibrosis distribution	Mesocardial, sparing the subendocardium, not in a coronary artery irroration territory	Linear	Thinned or normal	Enlarged LV
LHIAS	Elderly or middle age, female dominant	Interatrial septum, sparing the fossa ovalis	Transmural in the interatrial septum	Lobular	Thickened interatrial septum (usually >2 cm in transverse diameter)	Normal

ARVC: arrhythmogenic right ventricular cardiomyopathy; PILM: postmyocardial infarction lipomatous metaplasia; DCM: dilatative cardiomyopathy; LHIAS: lipomatous hypertrophy of the interatrial septum; MI: myocardial infarction; RV: right ventricle; LV: left ventricle; RVOT: right ventricular outflow tract. *Note.* Readapted from Kimura et al. [2].
